# Involvement of 8-*O*-acetylharpagide for *Ajuga taiwanensis* mediated suppression of senescent phenotypes in human dermal fibroblasts

**DOI:** 10.1038/s41598-020-76797-6

**Published:** 2020-11-12

**Authors:** Wei-Hsiang Hsu, Bing-Ze Lin, Jyh-Der Leu, Pin-Ho Lo, Hsueh-Yen Yu, Chao-Tsung Chen, Yuan-Heng Tu, Yun-Lian Lin, Yi-Jang Lee

**Affiliations:** 1 Department of Chinese Pharmaceutical Sciences and Chinese Medicine Resources, China Medical University, Taichung, 40402 Taiwan, ROC; 2 Department of Biomedical Imaging and Radiological Sciences, National Yang-Ming University, No. 155, Sec. 2, Linong St. Beitou District, Taipei, 11221 Taiwan, ROC; 3 Division of Radiation Oncology, Taipei City Hospital RenAi Branch, Taipei, 106 Taiwan, ROC; 4 Institute of Neuroscience, National Chengchi University, Taipei, 116 Taiwan, ROC; 5 Department of Traditional Chinese Medicine, Taipei City Hospital RenAi Branch, Taipei, 106 Taiwan, ROC; 6 Institute of Traditional Medicine, National Yang-Ming University, Taipei, 11221 Taiwan, ROC; 7 General Education Center, University of Taipei, Taipei, 11153 Taiwan, ROC; 8 Cancer Progression Research Center, National Yang-Ming University, Taipei, 11221 Taiwan, ROC

**Keywords:** Biochemistry, Cell biology, Drug discovery, Plant sciences, Biomarkers, Health care

## Abstract

Herbal medicines are attractive agents for human care. In this study, we found that the alcohol extract of *Ajuga taiwanensis* (ATE) screened from a chemical bank exhibited potent capacity for suppressing senescence associated biomarkers, including SA-β-gal and up-regulated p53 in old human dermal fibroblasts (HDFs) without induction of significant cytotoxicity up to 100 µg/ml. Concomitantly, cells re-entered the cell cycle by reducing G1 phase arrest and increasing cell growth rate. The ATE was further partitioned to obtain the sub-fractions of *n*-butanol (BuOH), ethyl acetate (EA) and water. The BuOH and water sub-fractions exhibited less effects on prohibition of cell growth than the EA sub-fraction. All of these sub-fractions exhibited the ability on suppressing SA-β-gal and p53 of old HDFs as low as 5–10 µg/ml. Under the activity guided fractionation and isolation, a major active constituent named AT-1 was isolated. The AT-1 was further identified as 8-*O*-acetylharpagide by structural analysis, and it could suppress SA-β-gal and p53 of old HDFs below 10 µM. In addition, the intracellular reactive oxygen species (ROS) levels of old HDFs were suppressed by ATE, the sub-fractions of BuOH and water, and AT-1. However, the EA sub-fraction showed little ability on suppression of ROS. Furthermore, we performed an in vivo study using aging mice to be fed with ATE and the sub-fractions followed by immunohistochemical (IHC) staining. The expression of p53 and SA-β-gal was significantly reduced in several tissue sections, including skin, liver, kidney, and spleen. Taken together, current data demonstrated that *A. taiwanensis* could suppress cellular senescence in HDFs, and might be used for health care.

## Introduction

Aging of human skin is an irreversible process that can be triggered by intrinsic disposition and extrinsic factors^1^. The dermis of human skin contains dermal fibroblasts that generate and control the formation of extracellular matrix (ECM) for maintenance of mechanical and structural properties of the skin^2^. How to ablate the senescence in dermal fibroblasts should be important for the field of rejuvenation science.

Senescent (aging) cells lose their ability to divide because of the limited quota of divisions^2^. Senescent cells exert an irreversible proliferative arrest and acidic β-galactosidase (β-gal) activity so called senescence-associated β-Gal (SA-β-Gal) accompanied by enlarged and flattened morphology^3^. Various cell stresses, including reactive oxygen species (ROS), DNA damage and oncogenes, could cause senescence^4^. ROS can cause damage to mitochondrial constituents, and, subsequently, damaged mitochondria produce more ROS. Besides, ROS could induce DNA damage and activate a DNA damage response, subsequently leading to activation of p53/p21^cip1^ pathway^5^. The activity of p53 is regulated through many mechanisms, such as SIRT1-p53-LDHA-Myc, p53–MDM2 axis, JNK/p53 etc. Activated p53 increases secretion of MCP-1 which is secreted as a major component of senescence associated secretory phenotype (SASP), which comprised of cytokines, matrix metalloproteinase, and growth factors^6–8^. In addition, p53 activation could induce cell growth arrest and apoptosis, as well as modulate cellular senescence and organismal aging^9^.

Herbal medicine has been reported to contain natural ingredients that would nourish life to delay or repress aging^10,11^. *Ajuga taiwanensis*, a folk medicine in Taiwan, has been used for several remedies, such as antitumor^12^, antimicrobes^13^, anti-inflammation^14^, antiarthritis ^15^, and chronic disorders^16^. Different kinds of active ingredients including phytoecdysteroids, neo-clerodane diterpenoids, anthocyanidin-glucosides, iridoid glycosides, flavonoids, and ergosterol-5, 8-endoperoxide^16–18^ have been isolated from the genus *Ajuga*. However, the potent ability of *A. taiwanensis* in anti-aging remains to be addressed.

In this study, the effects of *A. taiwanensis* on suppression of senescent related phenotypes in old human diploid fibroblasts (HDFs), including alcohol extracts (ATE), partitioned sub-fractions and a purified major compound were investigated in vitro and in vivo.

## Materials and methods

### Cell line, herbs and reagents

Human dermal fibroblasts (HDFs) were purchased from American Type Culture Collection (ATCC, Manassas, VA, USA) and cultured in Fibroblast Growth Kit-Low serum (ATCC PCS-201-041) containing 2% fetal bovine serum, 2 mM l-glutamate, and 50 U/ml penicillin (Sigma-Aldrich, St. Louis, MO, USA). Cells were incubated in a humidified incubator with 5% CO_2_ at 37 °C and passaged when they reached 80% confluence. *A. taiwanensis* was purchased from a local herbal retailer in Taipei, Taiwan, and taxonomically authenticated by I-Jung Lee, previous curator of the Herbarium of National Research Institute of Chinese Medicine (NRICM), Taipei, Taiwan, and the specimen was deposited in the Herbarium of NRICM.

### Preparation of the extracts of *A. taiwanensis* and following fractionation

The dried aerial parts of *A. taiwanensis* was pulverized and extracted with 80% ethanol at 60 °C overnight. The aqueous ethanol extract was concentrated under reduced pressure (Buchi Rotavapor R-200 at 50 °C) to obtain the crude *A. taiwanensis* extract (ATE). The crude extract was partitioned successively between water and ethyl acetate, and *n*-butanol (*v/v*, 1:1 each), respectively, to give ethyl acetate-(EA), *n*- butanol- (BuOH) and water- (H_2_O) soluble sub-fractions. Under the activity guided fractionation, one of the active fractions, ATE-BuOH was further purified over a Sephadex LH-20 (GE Healthcare Bio-sciences AB, Uppsala, Sweden) column using methanol as eluent repeatedly to yield the active compound (AT-1). The structure of the active compound was mainly characterized by 1D- and 2D-nuclear magnetic resonance (NMR) and mass spectrometry (MS) spectroscopic data. After concentration, each extract and pure compound were dissolved in dimethyl sulfoxide (DMSO) as stock solution or working solution.

### Cell proliferation

Cell proliferation was determined using the hemocytometry. The data were presented either by cell number or proliferation rate. The initial seeded cell number was regarded 100% for comparison with cell numbers obtained at different days.

### Flow cytometric analysis of the cell cycle

Cells were fixed in ice-cold 75% ethanol (10^6^ cells/3 ml) at 4 °C overnight. Subsequently, fixed cells were spun, treated with RNase A, centrifuged, and pellets were resuspended in propidium iodide (20 µg/ml) and subjected to flow cytometric analysis. The DNA contents were determined with the EPICS Profile (Coulter Electronics, Hialeah, FL, USA) and BD FACSCalibur system (BD, Franklin Lakes, NJ, USA) equipped with an air-cooled argon laser excited at 488 nm and analyzed by the CellQuest Pro software (version 5.1).

### Senescence associated-β-galactosidase (SA-β-gal) staining

Cells grown on the dishes were fixed in 0.2% glutaraldehyde/2% formaldehyde at 37 °C for 5 min. The staining solution (1 mg/ml of 5-Bromo-4-chloro-3-indolyl β-d-galactopyranoside, 5 mM potassium ferricyanide, 5 mM potassium ferrocyanide, 150 mM NaCl, 1 mM MgCl_2_, 40 mM citric acid (pH 6.0)) was used to treat fixed cells at 37 °C overnight. The stained cells could be visualized using the microscope with bright field (Olympus, Center Valley, PA, USA). For measurement of SA-β-gal activity, the Cellular Senescence Activity Assay kit (Cat# ENZ-KIT129) was used and the procedure was followed according to the manufacturer’s manual (Enzo Life Sciences Inc. Farmingdale, NY, USA)^19–21^. Briefly, the whole lysate of cells treated with cold 1 × cell lysis buffer was centrifuged for 10 min at 4 °C. Next, 50 μl of the supernatant was mixed with an equal amount of 2 × assay buffer and incubated at 37 °C for 1 h. The activity of SA-β-gal was then measured using a fluorescence plate reader at 360 nm (excitation)/465 nm (emission) (Multimode microplate readers TECAN 200/200Pro, TECAN Group Ltd., Männedorf, Switzerland).

### Immunofluorescence staining and confocal microscopy

Cells were seeded on cover slips for 48 h and fixed with 4% paraformaldehyde followed by 0.1% Triton X-100. The samples were blocked with 10% fetal bovine serum, and then incubated with anti-Ki-67 antibody (Millipore, Billerica, MA, USA). Nuclei were counterstained using 4′, 6-diamidino-2-phenylindole (DAPI). Cell images were acquired using a confocal fluorescence microscope (Leica TCS SP2, Leica Microsystems Ltd., Buffalo Grove, IL, USA).

### Western blot analysis

The Western blot analysis were followed by a previous report^22^. The primary antibodies were anti-p53 and anti-GAPDH (1:5000 each, Genetex Inc., Irvine, CA, USA).

### High performance liquid chromatography (HPLC)

Chemical profile analysis of ATE was run on a Hitachi 5160 Chromaster High-Performance Liquid Chromatograph (Hitachi, Palo Alto, CA, USA) equipped with a reverse-phase column (Purospher STAR RP-18 end capped, 5 µm, 150 × 4.6 mm; Merck Co., Kenilworth, NJ, USA) using a mobile phase of solvent A: 0.2% H_3_PO_4_ in H_2_O, solvent B: methanol with a program of 70% A for 5 min, 70–40% A for 15 min, 40–20% A for 10 min , and 20% A for 5 min. Solvent flow rate was set at 1 ml/min. Inject volume was 10 µl. The column oven was maintained at 40 °C and detected at UV 203 nm.

### Cell viability assay

MTT (3-(4, 5-Dimethylthiazol-2-yl)-2, 5-diphenyltetrazolium bromide) assay was used to evaluate the cell viability. In brief, 5 × 10^3^ cells per well were seeded in the 96-well plates. After treatment, each well was replaced with 0.5 mM MTT in serum free medium and incubated in the 37 °C incubator for 4 h. The mixtures were then replaced by dimethyl sulfoxide (DMSO) to solve the crystals, and the plates were placed in the Tecan’s Sunrise absorbance microplate reader (TECAN Group Ltd., Männedorf, Switzerland) for measurement of color intensity under 570 nm wavelength.

### Measurement of intracellular ROS

Intracellular reactive oxygen species (ROS) was measured using a Cellular ROS Detection Assay Kit (Cat#: ab113851, Abcam, Cambridge, UK). The kit contains 2′, 7′-Dichlorofluorescin diacetate (DCFDA), a cell permeable fluorogenic dye to measure hydroxyl, peroxyl and other ROS activity within the cell, according to the manufacturer's protocol. Briefly, cells were seeded in 96-well transparent bottom black-plate. After drug treatment for 24 h, the media was removed and 20 μM DCFDA was added to the corresponding wells and incubated for 30 min at 37 °C. The dye was washed away before reading. The signal was detected by the fluorescence spectroscopy with maximum excitation and emission spectra of 495 nm and 529 nm, respectively (Multimode microplate readers TECAN 200/200Pro, TECAN Group Ltd., Männedorf, Switzerland). Average relative fluorescence of control was equated to 100%, with treatment conditions calculated proportionally and signal was corrected by background signal and adjusted to the cell viability.

### Animal study and immunohistochemical (IHC) staining

Twelve months old Balb/C mice (N = 5) were used for evaluation of the effects of the ATE and the derived sub-fractions on suppression of p53, p16, cofilin-1 and SA-β-gal using the IHC analysis. Six-week old young mice (N = 5) was also purchased as the control without ATE treatment. The old mice were divided into pure water (control), ATE, ATE-BuOH and ATE-H_2_O. Each group was gastric tube fed twice a day and five days per week for a month. Tissues were resected from the animal and rinsed with PBS, fixed in 4% paraformaldehyde with gentle shaking at 4 °C overnight. The tissue sections were embedded in paraffin, and then de-paraffinized in xylene (Sigma-Aldrich, St. Louis, MO, USA) for 30 min followed by rehydrated in graded ethanol from 95%, 75% to 50% and finally in desterilized water or PBS. For antigen retrieval, tissues were boiled up in 1 mM EDTA Buffer (pH 8.0) for 20 min. The tissue sections were blocked with 5% H_2_O_2_ in goat serum. Tissue sections were incubated with anti-p53 (1:50; Millipore, Billerica, MA, USA), anti-p16^INK4^ (1:50; Cell Signaling Technology, Danvers, MA, USA), and anti-cofilin-1 (1:1000, Genetex Inc., Irvine, CA, USA) antibodies followed by the horseradish peroxidase (HRP)-conjugated secondary antibody (1:400; Sigma-Aldrich, St. Louis, MO, USA). The tissue sections were developed with 3′, 3′-diaminobenzidine (DAKO, Dako Denmark A/S Produktionsvej 42 DK-2600 Glostrup Denmark) and then counterstained with hematoxylin. Tissue sections were visualized under the optical microscope with a digital camera for acquisition of the images (BX61, Olympus, Center Valley, PA, USA). The animal study has been approved by Institutional Animal Care and Use Committee (IACUC) of National Yang-Ming University (No. 1031235). We have confirmed that all experiments were performed in accordance with relevant guidelines and regulations.

### Statistical analysis

Data were compared using two-way analysis of variant (ANOVA) or Student’s *t* test. Significant difference was defined as *p* < 0.05.

## Results

### Characterization of replicative senescence in human dermal fibroblasts

**Figure 1:**
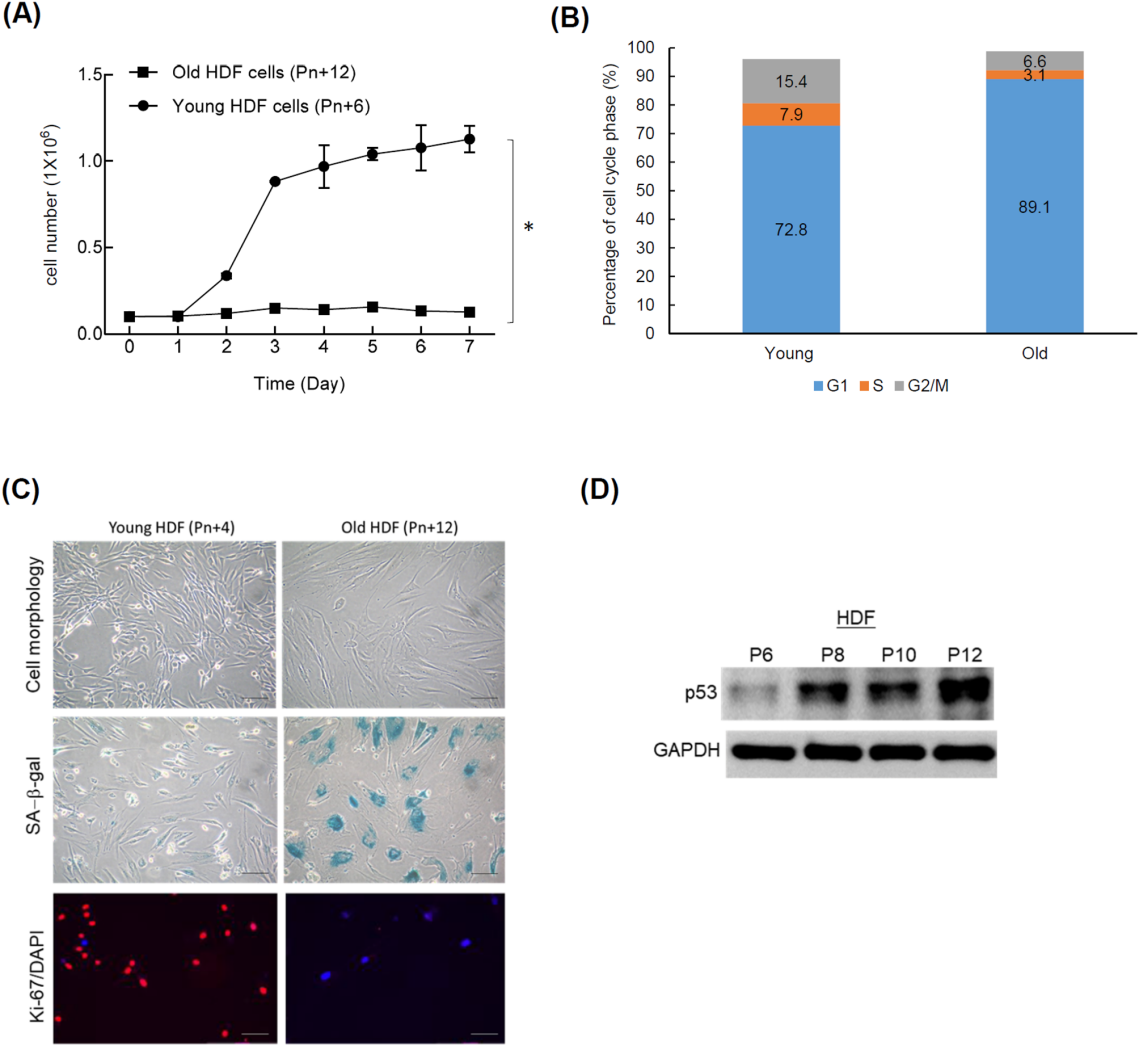
Cell aging analysis of human dermal fibroblasts (HDFs). (**A**) Different cell growth rates between young HDF and old HDF. (**B**) Increase of G1 and G2/M population but decrease of S population in old HDFs. (**C**) Comparison of cell morphology, SA-β-galactosidase, and Ki67 marker between young HDFs and old HDFs. Scale bar: 100 μm. (D) Detection of p53 expression in different passage number of HDFs. All experimental bio-repeats were more than 3 times, and data were represented as mean ± S.D. *p < 0.05.

We first demonstrated that the cell proliferation of old HDFs was significantly slower than that of young HDFs (Fig. 1A). The cell cycle analysis showed an apparent G1 phase arrest in old HDFs compared to young HDFs (Fig. 1B). The enlargement and flatten of cell morphology were accompanied by the increase of the SA-β-gal staining and the decrease of Ki-67 proliferation markers (Fig. 1C). SA-β-gal is a common biomarker of senescence, as it is a lysosomal enzyme that can be detected at pH 6.0 when lysosomal mass is increased in senescent cells but not young cells^3,23^. Furthermore, the expression of p53 was up-regulated in higher passage number of HDFs (Fig. 1D). We also found that cell cycle inhibitor p27^Kip1^ and p16^INK4^ were up-regulated in high passaged HDFs, although the levels were different (Supplementary Fig. S1). The following experiments focused on the expression of p53 and SA-β-gal staining to evaluate the effects of ATE.

### Effects of alcohol extract of *A. taiwanensis* (ATE) on cell viability and senescence suppression

**Figure 2:**
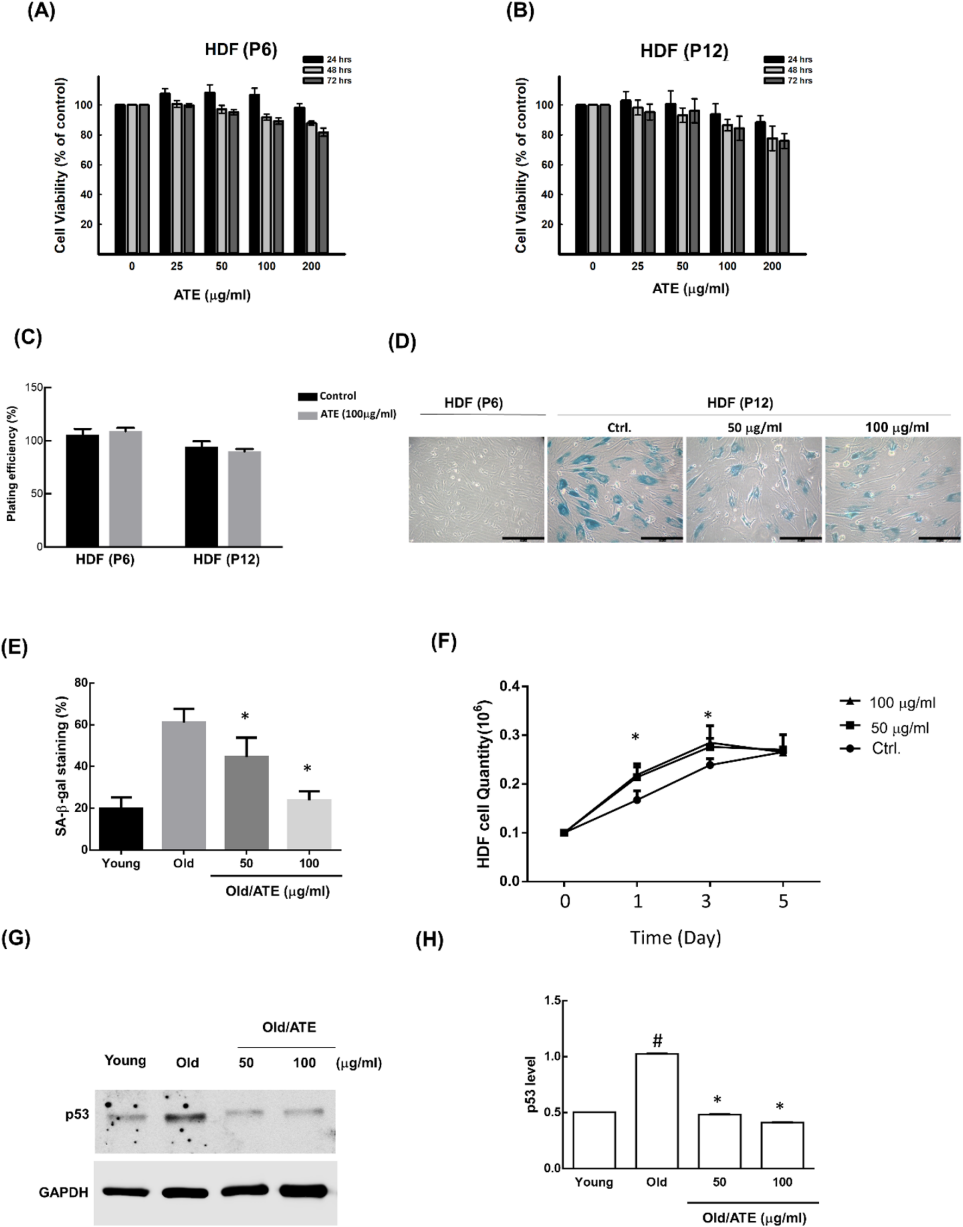
Effects of ATE on cytotoxicity and senescence of HDFs. (**A**) The MTT assay for examining the cytotoxicity of ATE on young HDFs within time course and dose-dependent experiments. (**B**) Examination of the cytotoxicity of ATE on old HDFs. (**C**) The colony formation assay for comparison of cell proliferative ability of young HDFs and old HDFs with or without the treatment of ATE. (**D**) Effects of ATE on the SA-β-gal staining of old HDFs. Scale bar: 100 μm. (**E**) Quantification of SA-β-gal expressive ratios in young HDFs and old HDFs with ATE treatment. (**F**) The cell proliferation analysis in old HDFs treated with different concentration of ATE. (**G**) Western blot analysis of p53 in old HDFs before and after treatment of ATE. (**H**) Densitometric measurement of p53 blots in HDFs treated with without ATE. All experimental bio-repeats were more than 3 times, and data were represented as mean ± S.D. *p < 0.05 compared to the untreated old controls. ^#^*p* < 0.05 compared to the young control.

We previously exploited a chemical bank provided by NRICM to seek if any novel herbal medicine would potentially cell senescence. The expression of SA-β-gal was used as a senescent marker for screening, and ATE was found to significantly reduce the level of SA-β-gal staining (data not shown). To examine if this effect was due to loss of cell viability, cells were treated with different concentrations of ATE for 24, 48 and 72 h followed by MTT assay. The results showed that 80% of cell viabilities remained detectable after both young HDFs and old HDFs were exposed to ATE up to 200 μg/ml (Fig. 2A,B). The plating efficiency of HDFs were also not affected by ATE up to 14-days of incubation using the clonogenic assay (Fig. 2C). We next investigated the level of SA-β-gal staining in HSFs before and after ATE treatment. The results showed that high level of SA-β-gal staining in old HDFs could be suppressed by ATE (Fig. 2D). The ratio of SA-β-gal suppression in cells treated with different concentrations of ATE was measured (Fig. 2E). The growth rate of old HDFs was increased up to 3 days of culture in the presence of ATE (Fig. 2F). The cell cycle analysis showed that old HDFs could re-enter the G1 phase after the treatment of ATE (Supplementary Fig. S2). Additionally, the up-regulated p53 in old HDFs was significantly suppressed by ATE using the Western blot analysis (Fig. 2G,H). Therefore, these results suggested that ATE could suppress the senescence of HDFs without induction of severe cytotoxicity.

### Effects of sub-fractions derived from ATE on suppression of cellular senescence

**Figure 3:**
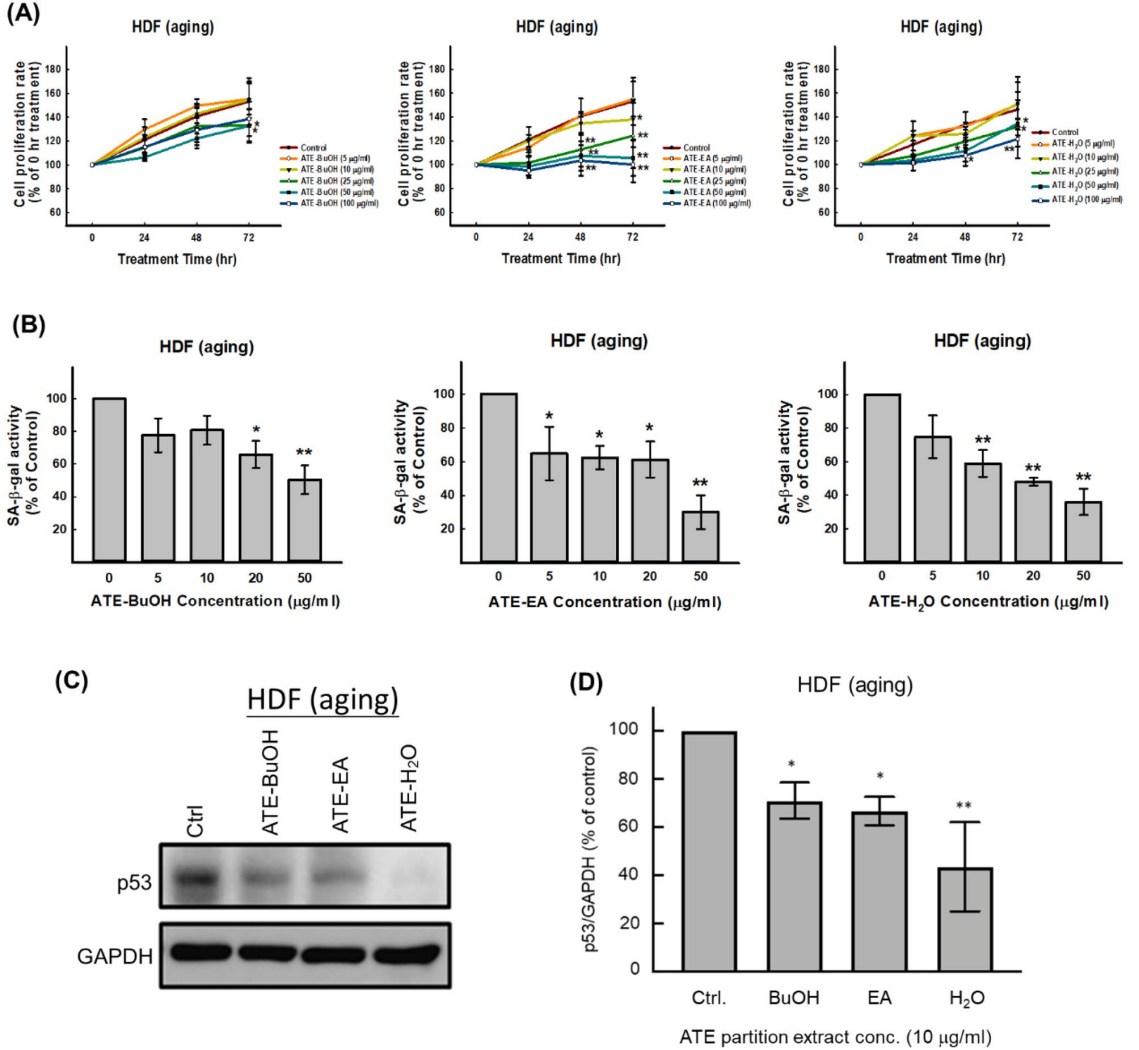
Effects of sub-fractions of ATE on cell proliferation and senescence of HDFs. (**A**) Comparison of cell proliferation in old HDFs treated with different concentrations of ATE sub-fractions up to 72 h. (**B**) Measurement of SA-β-gal activity in old HDFs treated with different concentrations of ATE sub-fractions for 48 h. (**C**) Western blot analysis for detection of p53 in old HDFs treated with different sub-fractions of ATE (10 μg/ml for each sub-fraction) for 48 h. (**D**) Densitometric quantification of the p53 blot. All experimental bio-repeats were more than 3 times, and data were represented as mean ± S.D. *p < 0.05; **p < 0.01 compared to the control (Ctrl).

The ATE was further partitioned to obtain different sub-fractions of ethyl acetate (EA), *n*-butanol (BuOH) and H_2_O-soluble. These sub-fractions showed various effects on cell proliferation of old HDFs using different concentrations up to 200 μg/ml. The results displayed that 100 µg/ml BuOH, EA, and H_2_O sub-fractions decreased about 18%, 35%, and 21% cell viability, respectively, at 72 h. Though the BuOH and H_2_O sub-fractions exhibited less inhibitory effect than the EA sub-fraction at high concentrations (50 and 100 µg/ml) (Fig. 3A). We found that BuOH and H_2_O sub-fractions did not affect cell proliferation, but EA sub-fraction has significant cytotoxicity from 10 µg/ml at 72 h. On the other hand, all of these sub-fractions did not significantly influence the cell proliferation of young HDFs (Supplementary Fig. S3). These sub-fractions exhibited the abilities on suppression of the SA-β-gal activity in old HDFs. The lowest concentrations of BuOH, EA, and H_2_O sub-fraction for significantly suppressing SA-β-gal activity were 20, 5, and 10 µg/ml, respectively (Fig. 3B). Additionally, the expression of p53 in old HDFs was concomitantly suppressed by these sub-fractions after 48 h of treatment, and it appeared that the H_2_O sub-fraction was more effective in suppressing p53 expression than the other two sub-fractions (Fig. 3C,D). These results imply that potent active ingredients may be existed in ATE for against p53 associated cellular senescence.

### A major compound isolated from BuOH and identified in H_2_O sub-fraction of ATE for against senescent associated biomarkers

**Figure 4:**
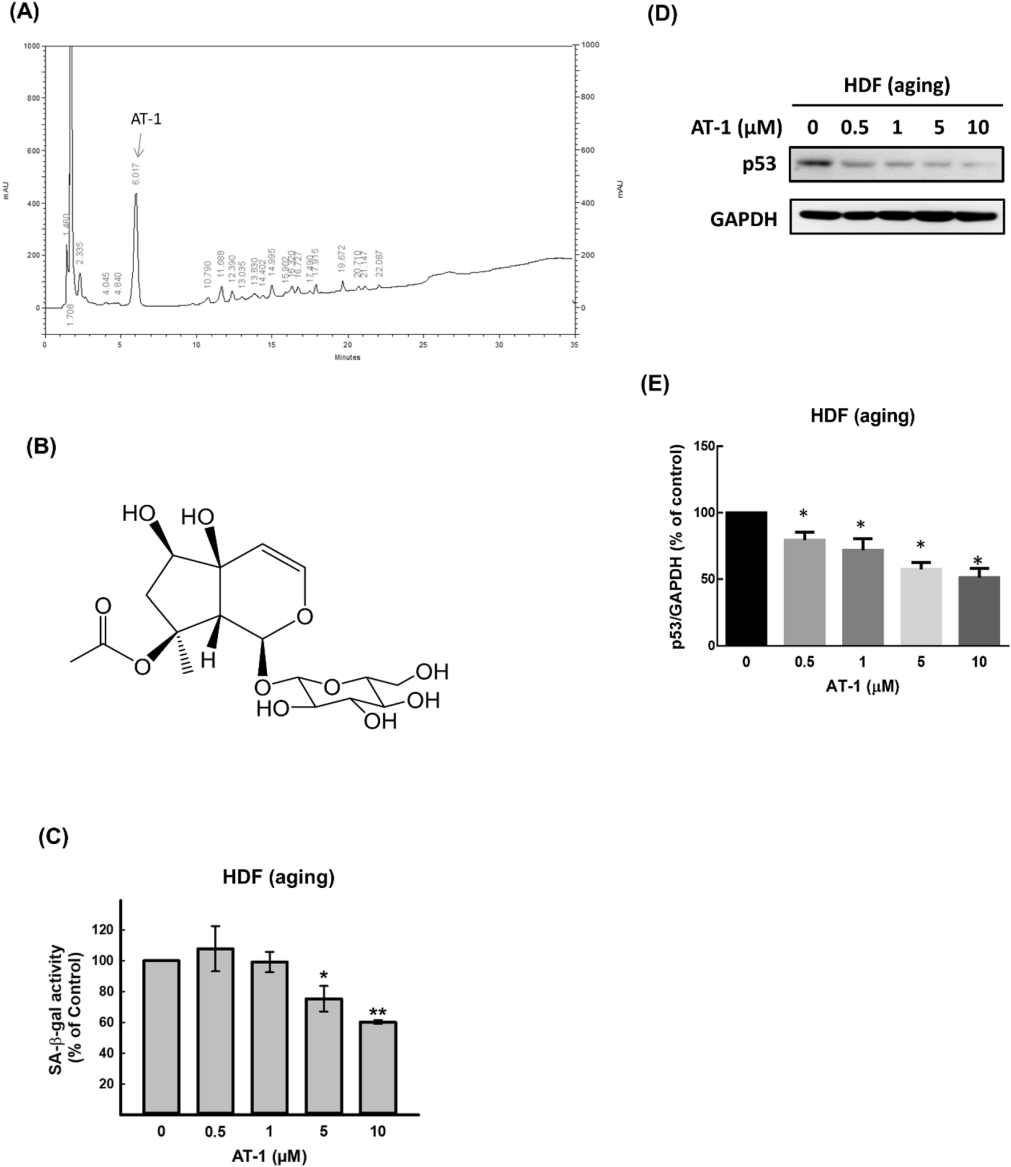
Purification of an active compound from ATE. (**A**) Analysis of the chemical profile of ATE by HPLC. The active compound was named as AT-1. (**B**) The structure of active compound (AT-1). (**C**) Suppression of SA-β-gal activity by AT-1 in old HDFs. (**D**) Suppression of p53 expression by AT-1 in old HDFs. (**E**) Densitometric quantification of the p53 blot. All experimental bio-repeats were more than 3 times, and data were represented as mean ± S.D. *p < 0.05 and **P < 0.01 compared to the control group.

We further used column chromatography to purify the active components in BuOH fraction (see “Materials and methods”). A major active component (AT-1) was isolated and detected as shown in the chemical profile (Fig. 4A). The structure of AT-1 was to be 8*-O*-acetylharpagide by extensive 1D- and 2D-NMR and MS spectroscopic data (Fig. 4B). For 48 h of treatment, AT-1 could suppress the activity of SA-β-gal in old HDFs up to 10 μM (Fig. 4C). Concomitantly, the expression of p53 was suppressed by AT-1 with a dose-dependent manner (Fig. 4D,E). These results suggest that AT-1 named 8-O-acetylharpagide is an important component of ATE for against senescent phenotypes, at least in part.

### Effects of ATE, derived sub-fractions, and AT-1 active compound on regulation of ROS level in HDFs

**Figure 5:**
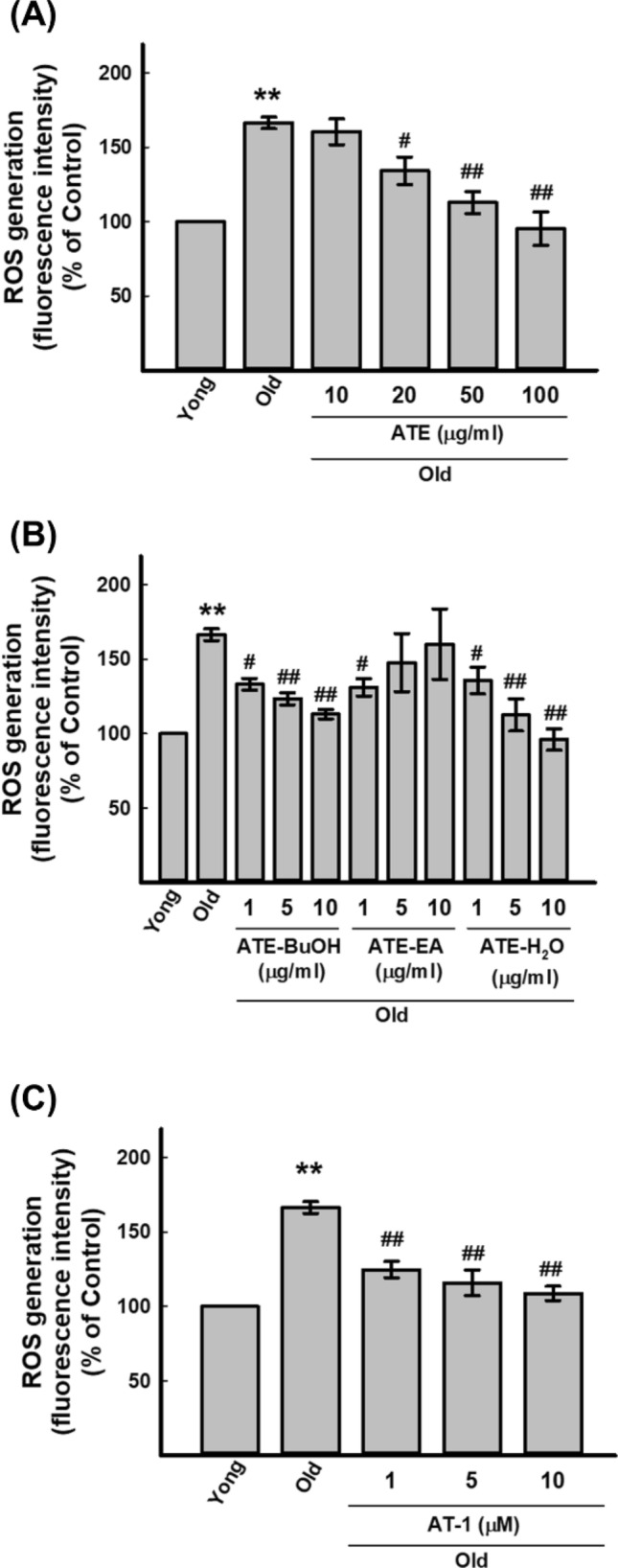
Measurement of the ROS levels in HDF cells. (**A**) Effects of ATE on the ROS generation of old HDFs. (**B**) Effects of different sub-fractions of ATE on the ROS generation of old HDFs. (**C**) Effects of AT-1 on the ROS generation of old HDFs. All of the treated cells were compared to untreated controls. All experimental bio-repeats were more than 3 times, and data were represented as mean ± S.D. * *p* < 0.05, ** *p* < 0.01 compared to control; ^#^*p* < 0.05, ^##^*p* < 0.01 compared with old cells at the same incubation time.

We next investigated if ATE could affect the intracellular ROS level in HDFs. The DCFDA method was used for measuring the change of intracellular ROS after treatments (see “Materials and methods”). The results showed that a concentration-dependent suppression of ROS was detected in ATE treated old HDFs (Fig. 5A). For different sub-fractions of ATE, both BuOH and H_2_O sub-fractions also exhibited the similar effects with ATE, but the EA sub-fraction only suppressed ROS of old HDFs at 1 µg/ml (Fig. 5B). Furthermore, the AT-1 active compound showed similar effects on suppression of ROS from low (1 µM) to high (10 µM) concentrations (Fig. 5C). Therefore, ATE could suppress the generation of ROS in old HDFs, and this effect might be associated with the effects of ATE on the expression of SA-β-gal and p53.

### Effects of ATE and the derived sub-fractions on tissues of aging mice

**Figure 6:**
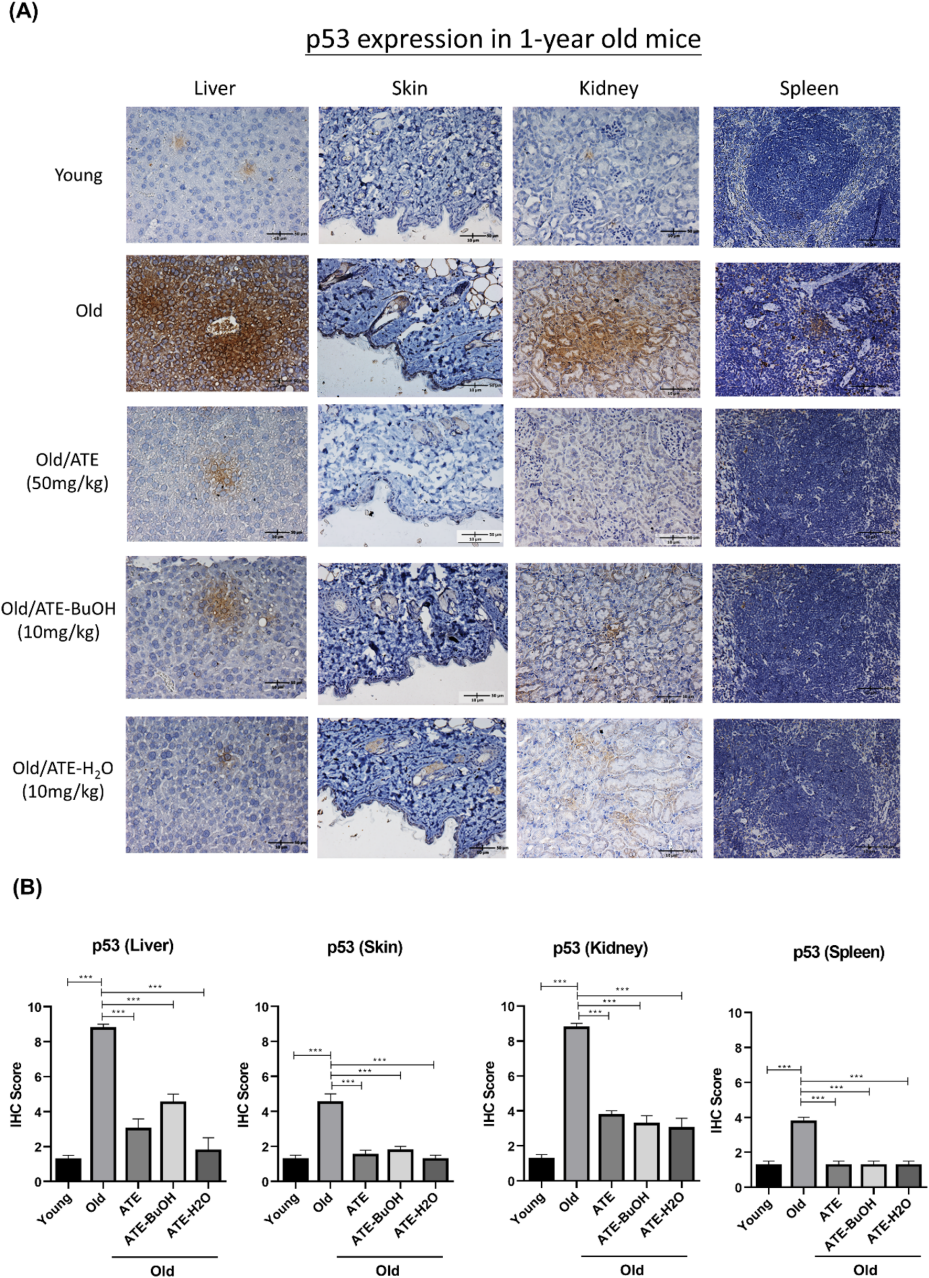

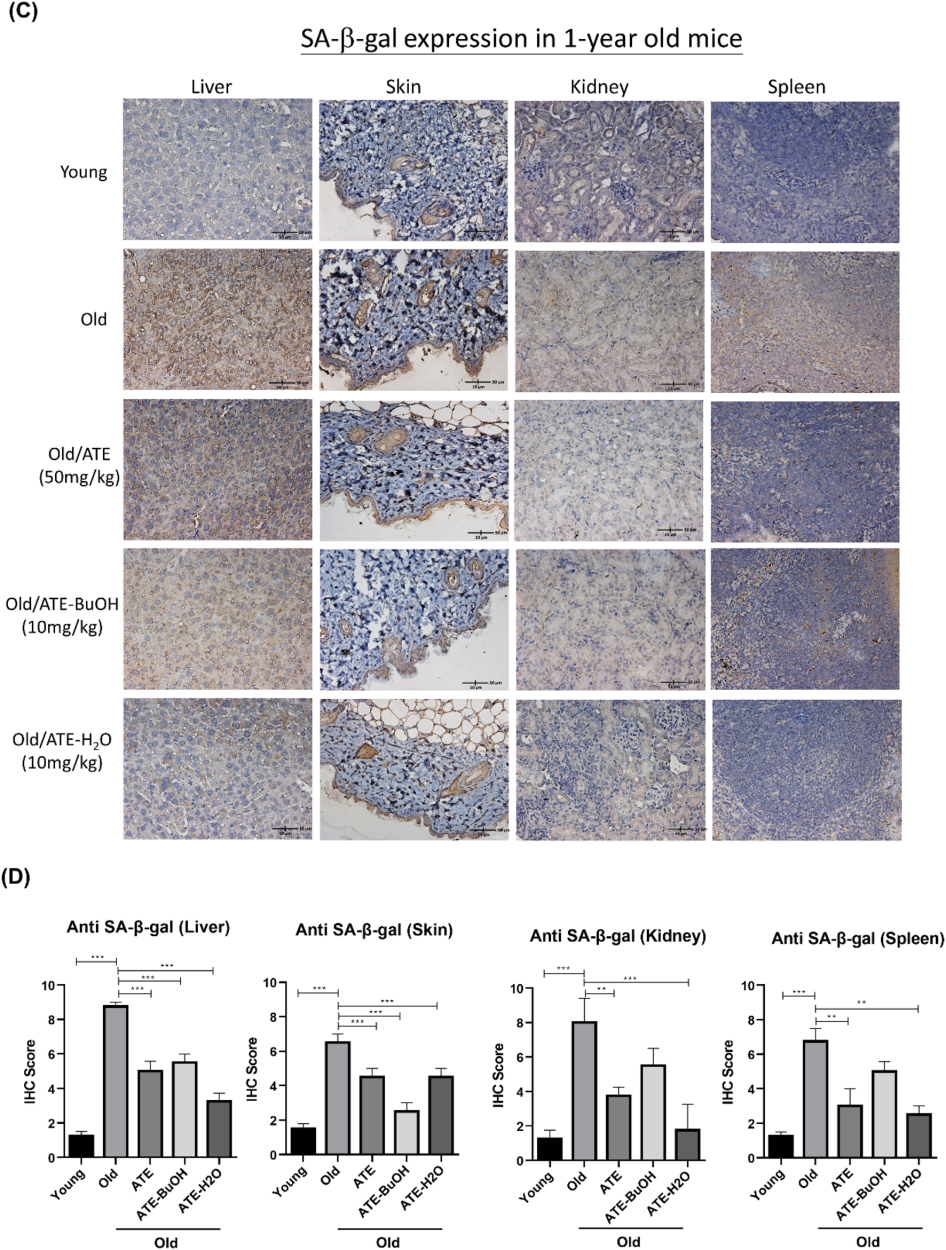
Effects of ATE on the expression of p53 in vivo. (**A**) IHC examination of p53 in aging mice with or without the treatment of ATE and partitioned sub-fractions. (**B**) Comparison of p53 expression in tissues by the percentage of IHC scores. (**C**) IHC examination of SA-β-gal. (**D**) Comparison of SA-β-gal expression in tissues by the percentage of IHC scores. The expressions of p53 and SA-β-gal were represented by the brown stains. All experimental bio-repeats were more than 3 times, and data were represented as mean ± S.D. **p < 0.01; ***p < 0.001.

**Figure 7:**
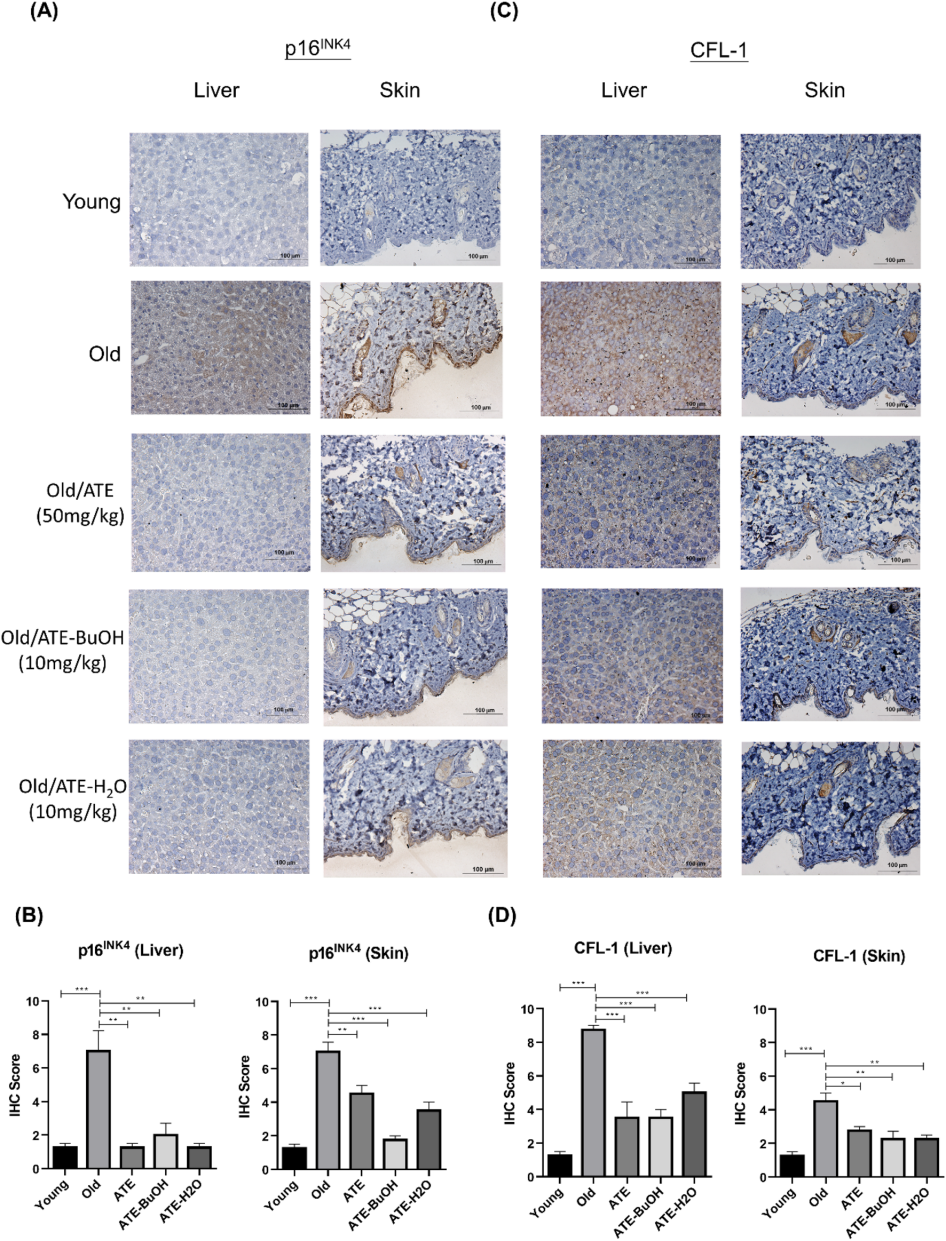
Effects of ATE on the expression of p16^INK4^ and CFL-1 in vivo. (**A**) IHC examination of p16^INK4^ in liver tissues and skin tissues of aging mice with or without the treatment of ATE and partitioned sub-fractions. (**B**) Quantification of p16^INK4^ level by IHC scores. (**C**) IHC results of CFL-1 expression in tissues. (**D**) Quantification of CFL-1 level by IHC scores. All experimental bio-repeats were more than 3 times, and data were represented as mean ± S.D. ***p < 0.001.

We next investigated the effects of ATE and the sub-fractions of BuOH and H_2_O on aging mice (see “Materials and methods”). The sub-fraction of EA was omitted because it showed a stronger effect on suppression of cell proliferation compared to another sub-fractions (Fig. 3A). The IHC staining of liver, skin, kidney and spleen tissues showed that ATE and the sub-fractions could decrease the expressions of p53 compared to the water-fed control group (Fig. 6A). The results were also quantified by the IHC scores that revealed the significant suppression of p53 level in aged tissues after treatment (Fig. 6B). The expression of SA-β-gal was also detected by IHC staining for these tissues. The level of this biomarker was suppressed in aged tissues treated with ATE and sub-fractions, although the effect of BuOH sub-fraction on kidney and spleen tissues was less significant (Fig. 6C,D). In addition, we also found that p16^INK4^ expressed in liver and skin tissues of aging mice was down-regulated by ATE and sub-fractions of BuOH and H_2_O (Fig. 7A,B). We also detected the expression of cofilin-1 (CFL-1) that had been reported to be accumulated in aging tissues^24^. The results showed that ATE and both sub-fractions could reduce the CFL-1 level in both liver and skin tissues (Fig. 7C,D). This in vivo study suggests that ATE may potentially regulate the aging of tissues.

## Discussion

Anti-aging of skin is the most attractive topic because it is related to dramatic cosmetic markets and economies. The primary strategy of anti-aging agents is to provide deep moisturizing effects, replenishment of depleted compartments, anti-oxidant capacities, renewal stimulation of the skin, and the natural homeostatic maintenance linked to healthy of dermal fibroblasts^24^. However, chemical or growth factor based anti-aging compounds may raise unexpected side effects^25^. On the other hand, the safety of herbal medicine used for designing of cosmetic products is highly acceptable^26,27^. In this study, high passage of HDFs exhibited delayed cell growth rate accompanied by increase of p53 and SA-β-gal. ATE and the derived sub-fractions and potent active compound AT-1 could suppress both senescent related biomarkers by current data. A critical role of p53 on senescence and aging has been reported, including the skin keratinocytes^9,28^. Although the influence of p53 on aging is still not fully understood, increase of p53 level is still regarded an important marker of aging. The shortening of telomeres and accumulation of DNA damage are probably the cause of p53 up-regulation^29,30^.

In addition to ATE, the partitions of ATE were also isolated and used to compare their effects with ATE. It revealed that lower concentration of all sub-fractions could suppress p53 and SA-β-gal in old HDFs compared to crude ATE, suggesting that certain active ingredient(s) might exist in these sub-fractions for suppression of the cellular senescence. Although EA sub-fraction could suppress SA-β-gal activity, it also exhibited higher cytotoxicity than the other two sub-fractions. The effects of BuOH and H_2_O sub-fractions on suppression of p53 and SA-β-gal under the same concentration are more consistent. Indeed, the ROS levels of old HDFs were suppressed after the treatment of BuOH and H_2_O sub-fractions but not EA sub-fraction. A purified active compound, 8-*O*-acetylharpagide (AT-1) was isolated from *n*-BuOH and identified in H_2_O sub-fraction, which is the major constituent according to the HPLC analysis. 8-*O*-acetylharpagide belongs to iridoid glycosides, which has been reported to be isolated from other genus *Ajuga* with the activities of vasoconstriction, anti-inflammation, anti-bacteria, and anti-virus^31,32^. Here we provided an evidence that the low concentration (below 10 µM) of 8-*O*-acetylharpagide could sufficiently suppress senescence related phenotypes in old HDFs. This evidence-based study suggests that the extract of *A. taiwanensis* possesses anti-aging activity that may be related to the high percentage of 8-*O*-acetylharpagide in this type of herbal medicine.

We have noticed that under the same low concentration of 8-*O*-acetylharpagide treatment (0.5–1 µM), down-regulation of p53 was more significant than that of SA-β-gal (Fig. 4). It implies that this compound may influence the expression of p53 prior to suppression of senescence under low concentration. Little is known whether 8-*O*-acetylharpagide can directly regulate p53. A recent publication reports that Romanian *A. genevensis* L. and *A. reptans *L. (Lamiaceae) harbor anti-oxidant activity, and 8-*O*-acetylharpagide is one of the active compounds in the phytochemical profiles^33^. Because the oxidative stress is able to up-regulate p53, 8-*O*-acetylharpagide mediated down-regulation of p53 may be associated with ablation of oxidative stress. However, this hypothesis remains to be addressed in the future.

We also performed in vivo experiments to demonstrate the physiological activity of ATE. Oral administration was exploited because this approach has been applied in rat and rabbit model^32^. We exploited IHC to examine the expression of p53, SA-β-gal, p16^INK4^, and CFL-1 in various tissues of aging mice as a pilot study. It appeared that these senescence associated biomarkers could be suppressed by ATE and sub-fractions of BuOH and water in selected tissues. A recent report has shown that the up-regulation of p16^INK4^ transcript was lowest in skin (1.9-fold) compared to liver (12.6-fold) and all other tissues of aged mice^34^. However, the expression of p16^INK4^ remains higher in skin tissues of old mice compared to that of young mice, and ATE could suppress this biomarker in old skin tissue according to current data. Besides, we and others have found that CFL-1 is also associated with cell senescence (^24^ and manuscript under revision), although the role of CFL-1 in cell senescence remains to be addressed. Suppression of CFL-1 by ATE and its sub-fractions would be of interest to be investigated in the future.

In summary, current data demonstrated that aqueous ethanol extract of *A. taiwanensis* could suppress SA-β-gal and p53 of old HDFs at a concentration with little cytotoxicity. This activity was not only found in crude extract but also in BuOH and water sub-fractions that raised the similar effect at even lower concentration. The major constituent 8-*O*-acetylharpagide was identified in the sub-fractions, and might play an important role for this anti-senescent activity. To the best of our knowledge, this is the first report proposing that the extract of *A. taiwanensis* contains the active compound to regulate the senescent related markers in vitro and in vivo. It may contribute to design of novel anti-aging strategies.

## Supplementary information

Supplementary Information.
