# Author Correction: Involvement of 8-*O*-acetylharpagide for *Ajuga taiwanensis* mediated suppression of senescent phenotypes in human dermal fibroblasts

**DOI:** 10.1038/s41598-025-90257-z

**Published:** 2025-02-19

**Authors:** Wei‑Hsiang Hsu, Bing‑Ze Lin, Jyh‑Der Leu, Pin‑Ho Lo, Hsueh‑Yen Yu, Chao‑Tsung Chen, Yuan‑Heng Tu, Yun‑Lian Lin, Yi‑Jang Lee

**Affiliations:** 1 Department of Chinese Pharmaceutical Sciences and Chinese Medicine Resources, China Medical University, Taichung, 40402 Taiwan, ROC; 2 Department of Biomedical Imaging and Radiological Sciences, National Yang-Ming University, No. 155, Sec. 2, Linong St. Beitou District, Taipei, 11221 Taiwan, ROC; 3 Division of Radiation Oncology, Taipei City Hospital RenAi Branch, Taipei, 106 Taiwan, ROC; 4 Institute of Neuroscience, National Chengchi University, Taipei, 116 Taiwan, ROC; 5 Department of Traditional Chinese Medicine, Taipei City Hospital RenAi Branch, Taipei, 106 Taiwan, ROC; 6 Institute of Traditional Medicine, National Yang-Ming University, Taipei, 11221 Taiwan, ROC; 7 General Education Center, University of Taipei, Taipei, 11153 Taiwan, ROC; 8 Cancer Progression Research Center, National Yang-Ming University, Taipei, 11221 Taiwan, ROC

Correction to: *Scientific Reports* 10.1038/s41598-020-76797-6, published online 12 November 2020

This Article contains an error in Figure 6 (A) where the image for ‘Old/ATE (50 mg/kg)’ was duplicated for ‘Old/ATE-BuOH (10 mg/kg)’.

The correct Figure 6 and accompanying legend appear below.

This change does not affect the conclusions of the Article.

**Fig. 6:**
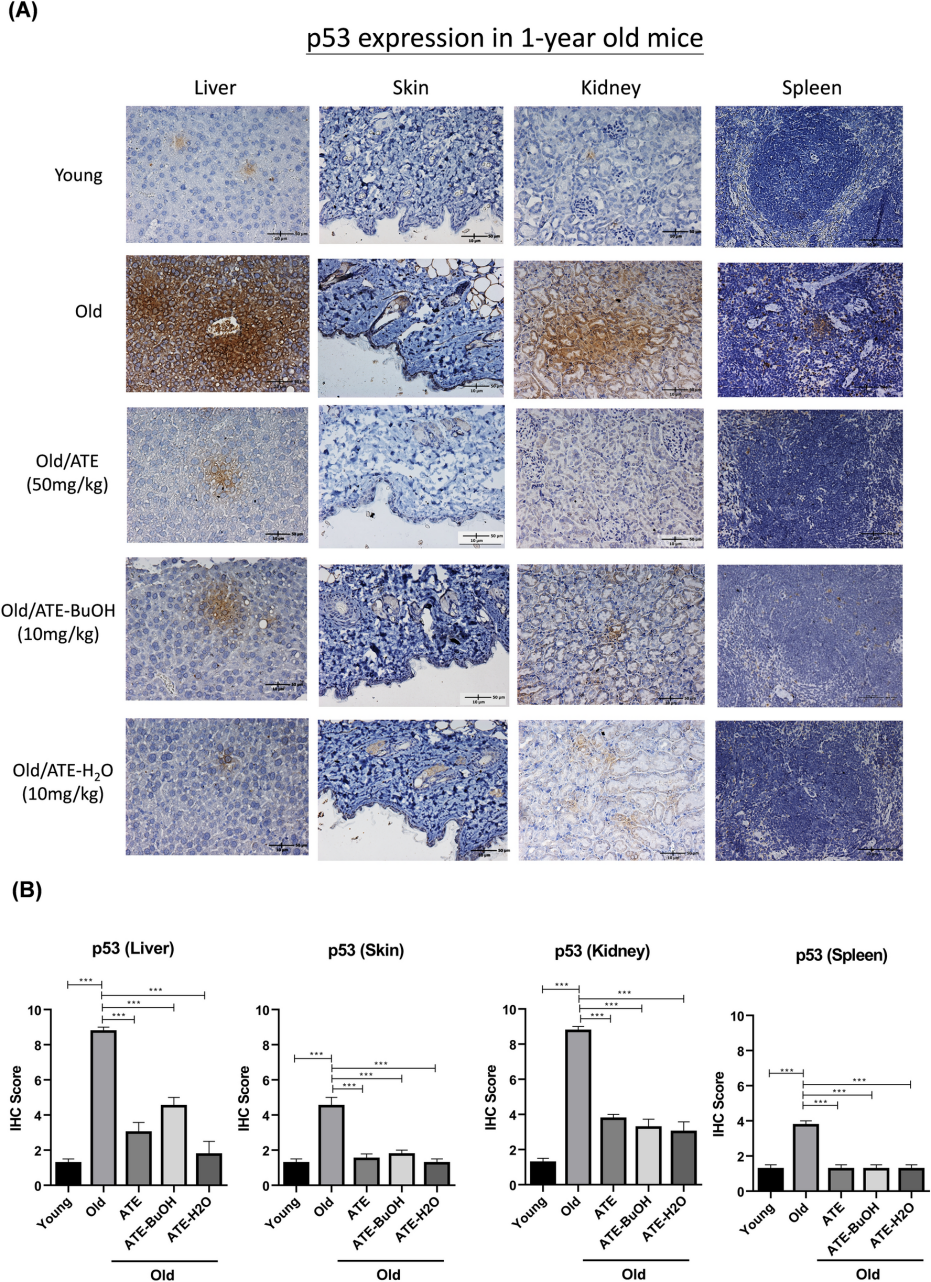
Effects of ATE on the expression of p53 in vivo. (**A**) IHC examination of p53 in aging mice with or without the treatment of ATE and partitioned sub-fractions. (**B**) Comparison of p53 expression in tissues by the percentage of IHC scores. (**C**) IHC examination of SA-β-gal. (**D**) Comparison of SA-β-gal expression in tissues by the percentage of IHC scores. The expressions of p53 and SA-β-gal were represented by the brown stains. All experimental bio-repeats were more than 3 times, and data were represented as mean ± S.D. **p < 0.01; ***p < 0.001.

