# Supplementary material for: Involvement of 8-O-acetylharpagide for Ajuga taiwanensis mediated suppression of senescent phenotypes in human dermal fibroblasts
Source: Sci Rep. 2020 Nov 12;10:19731. doi: 10.1038/s41598-020-76797-6 (PMC7661503; doi:10.1038/s41598-020-76797-6)
Supplement: Supplementary file 1 — Supplementary Information. [file 41598_2020_76797_MOESM1_ESM.pdf]

Involvement of 8-*O*-acetylharpagide for *Ajuga taiwanensis* mediated suppression of senescent phenotypes in human dermal fibroblasts

Wei-Hsiang Hsu[1,#], Bing-Ze Lin[2,#], Jey-Der Leu[3,4,#], Pin-Ho Lo[2#], Hsueh-Yen Yu[2], Chao-Tsung Chen[5,6,7], Yuan-Heng Tu[2], Yun-Lian Lin[1,*] and Yi-Jang Lee[2,8,*]

[1] Department of Chinese Pharmaceutical Sciences and Chinese Medicine Resources, China Medical University, Taichung 40402, Taiwan

[2] Department of Biomedical Imaging and Radiological Sciences, National Yang-Ming University, Taipei 11221, Taiwan

[3] Division of Radiation Oncology, Taipei City Hospital RenAi Branch, 106 Taipei

[4] Institute of Neuroscience, National Chengchi University, Taipei 116, Taiwan

[5] Department of Traditional Chinese Medicine, Taipei City Hospital RenAi Branch, 106 Taipei, Taiwan

[6] Institute of Traditional Medicine, National Yang-Ming University, 112 Taipei, Taiwan

[7] General Education Center, University of Taipei, Taipei, Taiwan

[8] Cancer Progression Research Center, National Yang-Ming University, Taipei 11221, Taiwan

#: These authors contributed equally.

*Address for correspondence:

Yun-Lian Lin, Ph.D.

China Medical University, Taichung, Taiwan

Department of Chinese Pharmaceutical Sciences and Chinese Medicine Resources

Tel: +886-4-22053366 ext 5520

E-mail: yllin@mail.cmu.edu.tw

Yi-Jang Lee, Ph.D.

Department of Biomedical Imaging and Radiological Sciences

National Yang-Ming University,

No. 155, Sec. 2, Linong St. Beitou District, 112, Taipei, Taiwan

Tel: 886-2-28267189, Fax: 886-2-28201095. E-mail: yjlee2@ym.edu.tw

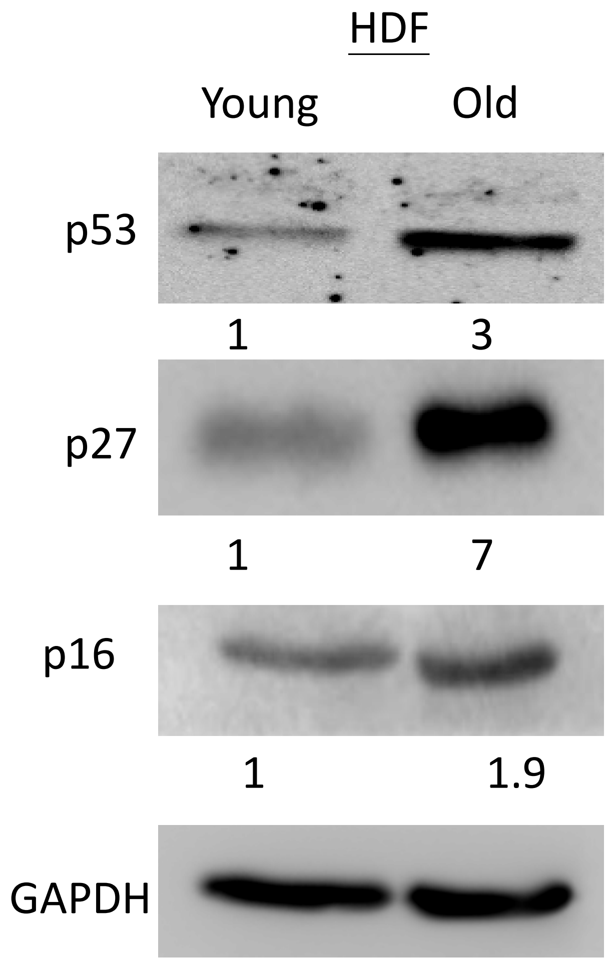

Supplementary Figure 1. Western blot analysis of senescent related proteins in HDFs.

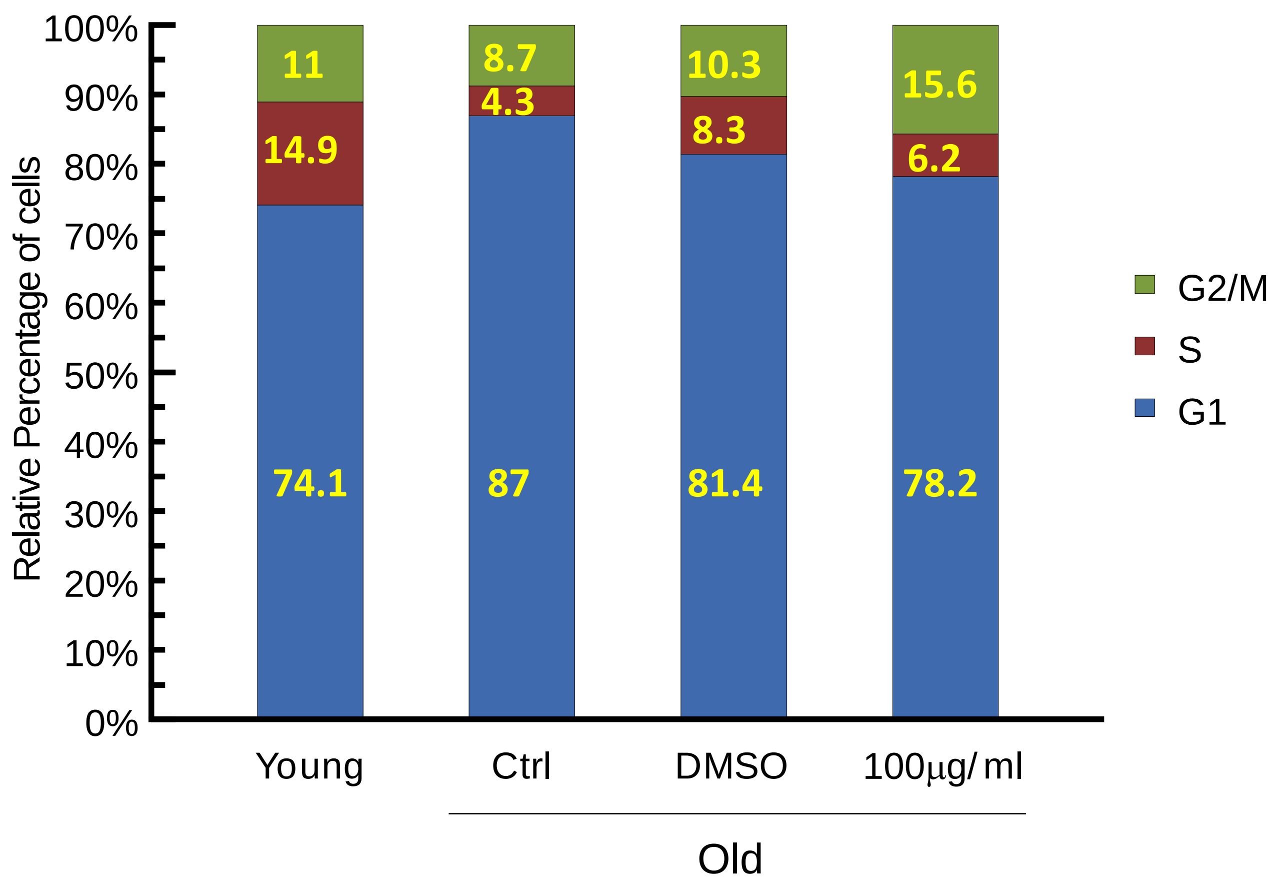

Supplementary Figure 2. Cell cycle distribution of HDFs. Old HDF were treated with ATE and compared to untreated controls and young HDF.

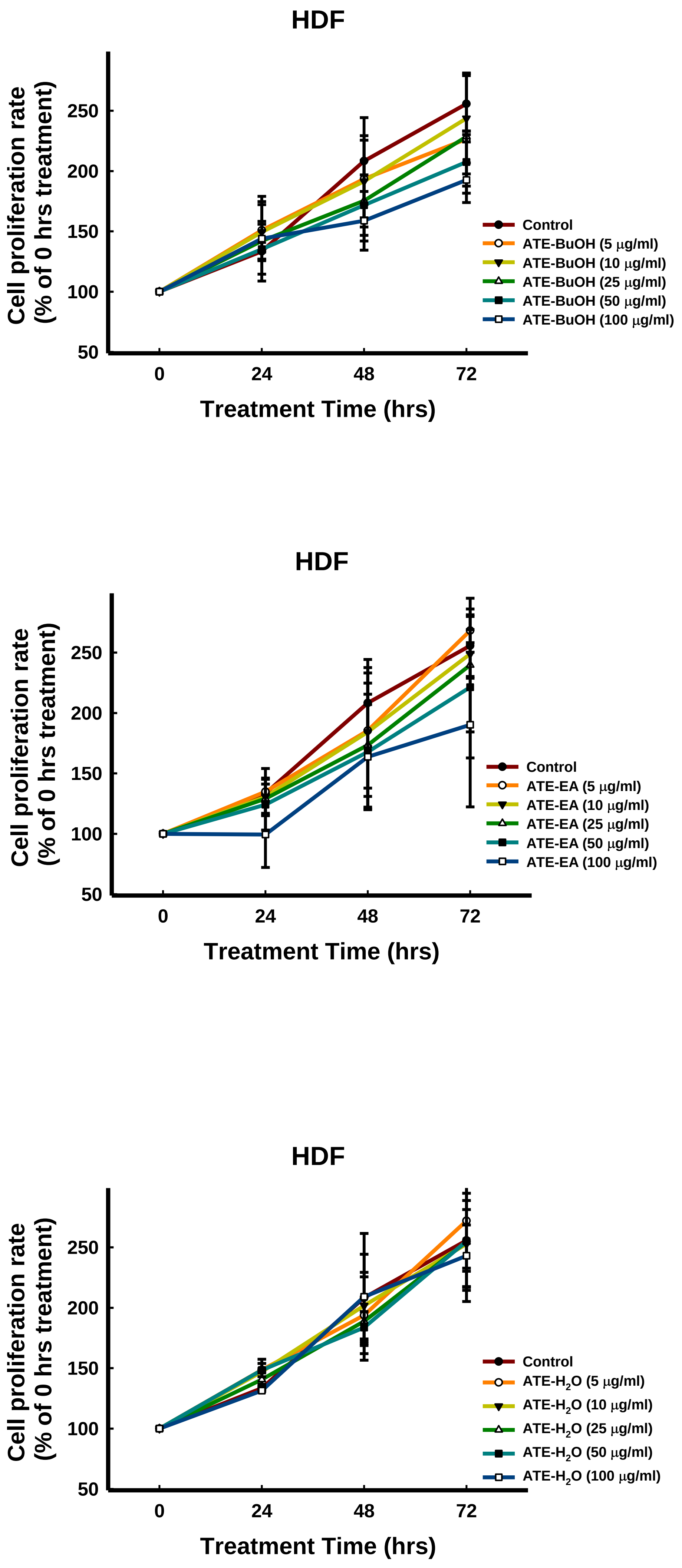

Supplementary Figure 3. Effects of different ATE sub-fractions on the cell proliferation rate of young HDFs.

Original gel and blots for each Western blot analysis

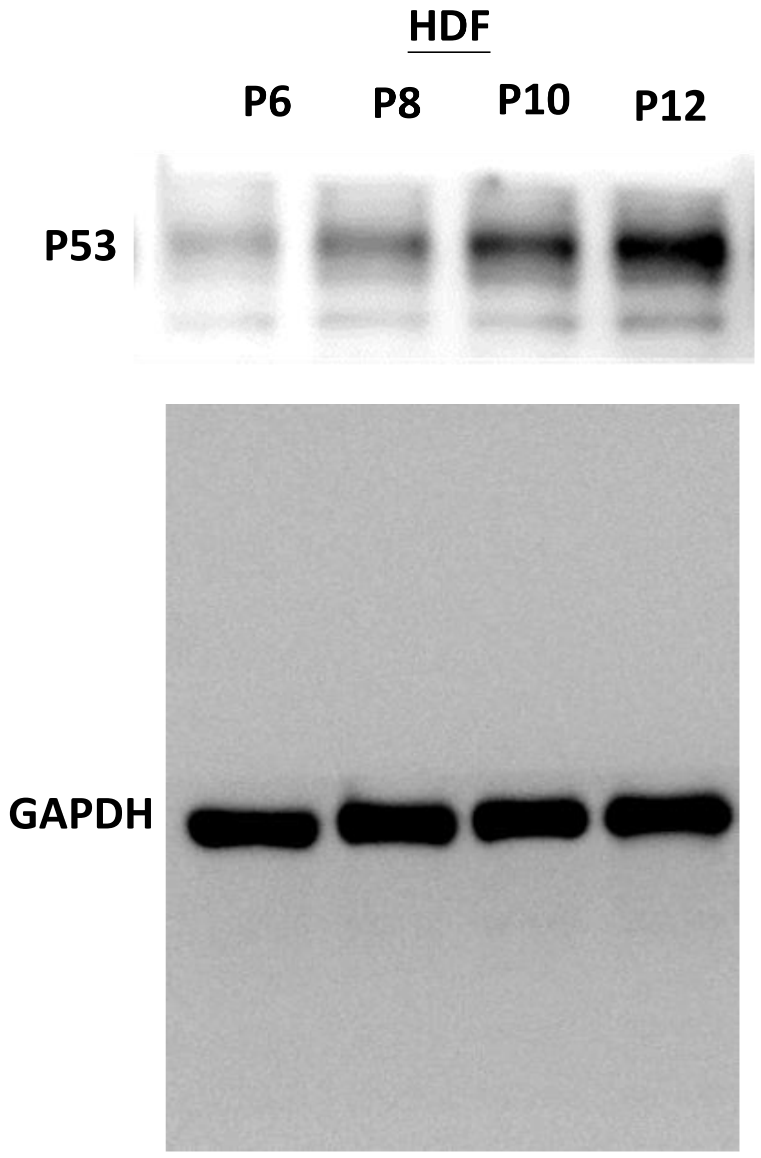

Figure 1D. Detection of p53 expression in different passage number of HDFs.

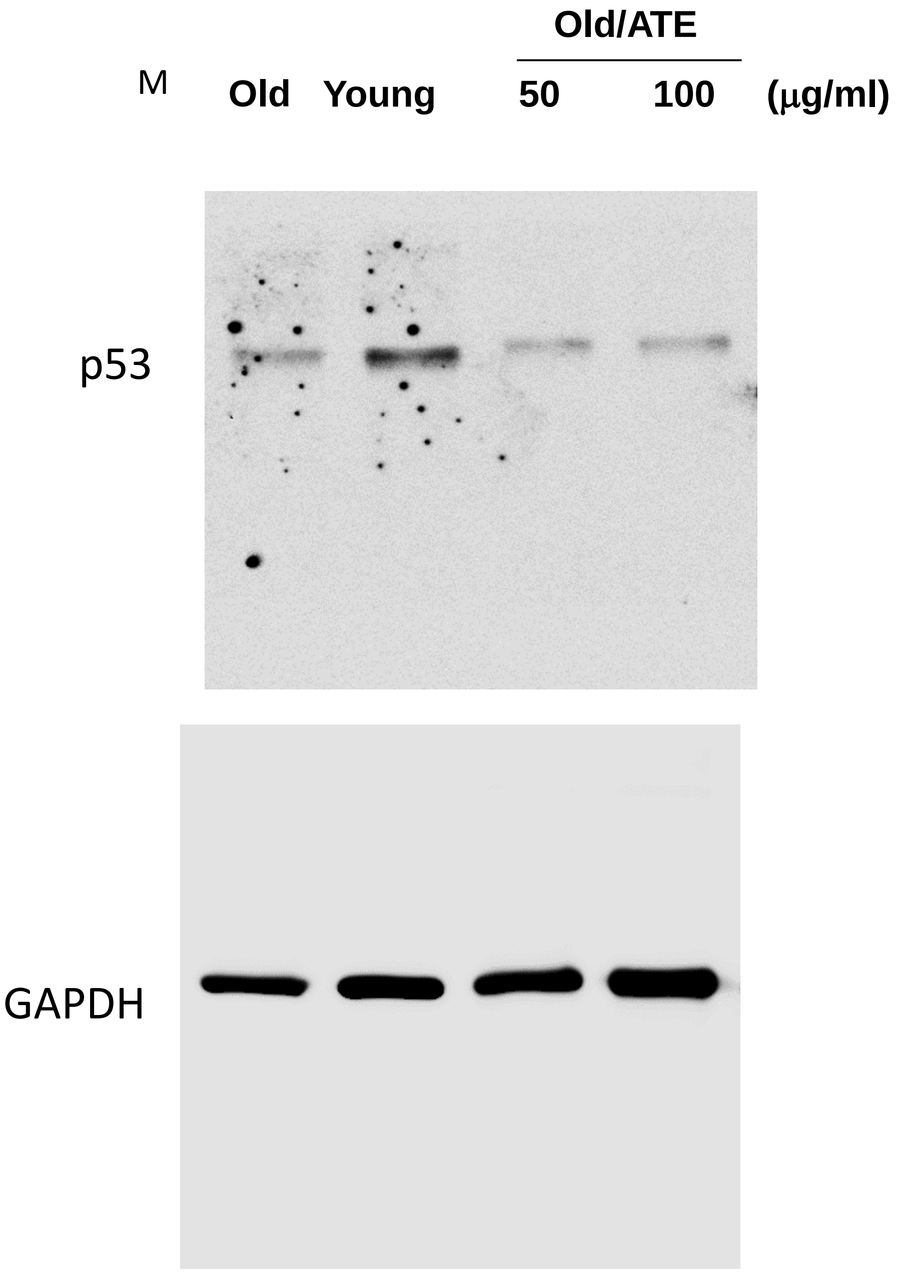

Figure 2G Western blot analysis of p53 in old HDFs before and after treatment of ATE.

Original gel and blots for each Western blot analysis

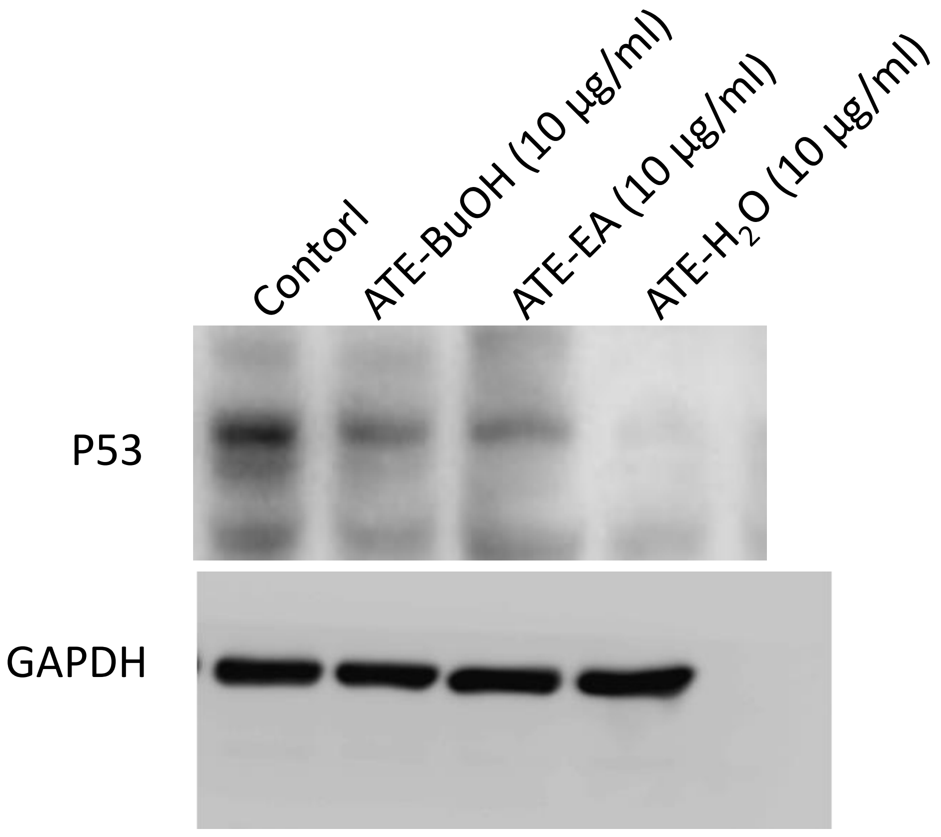

Figure. 3C Western blot analysis for detection of p53 in old HDFs treated with different sub-fractions of ATE (10mg/ml for each sub-fraction) for 48 hours.

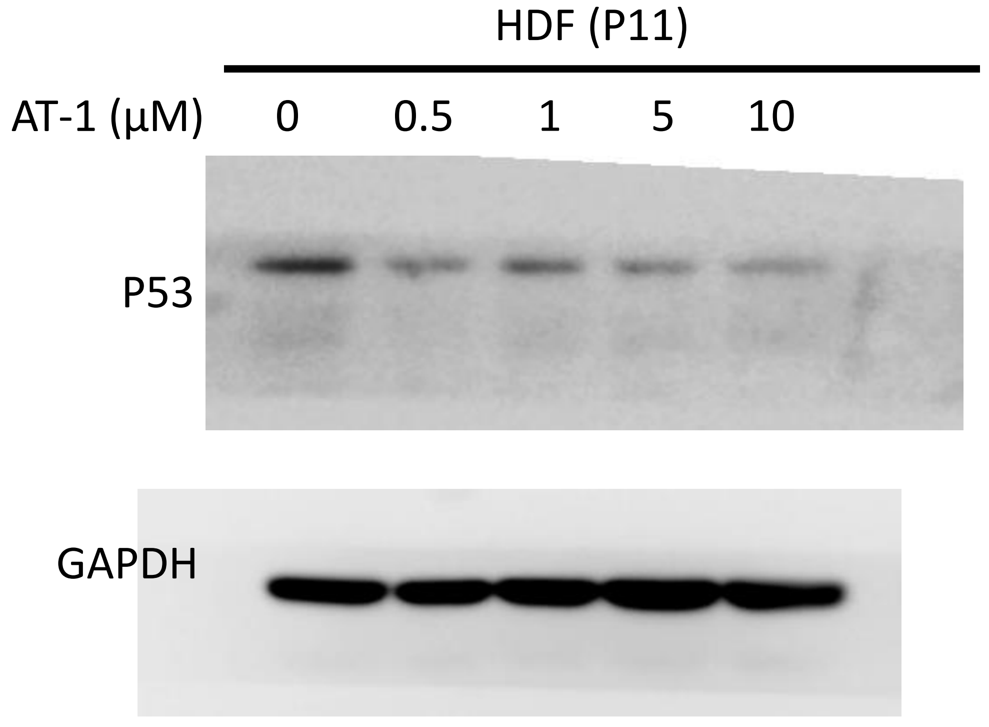

Fig. 4D Suppression of p53 expression by AT-1 in old HDFs.

Original gel and blots for each Western blot analysis

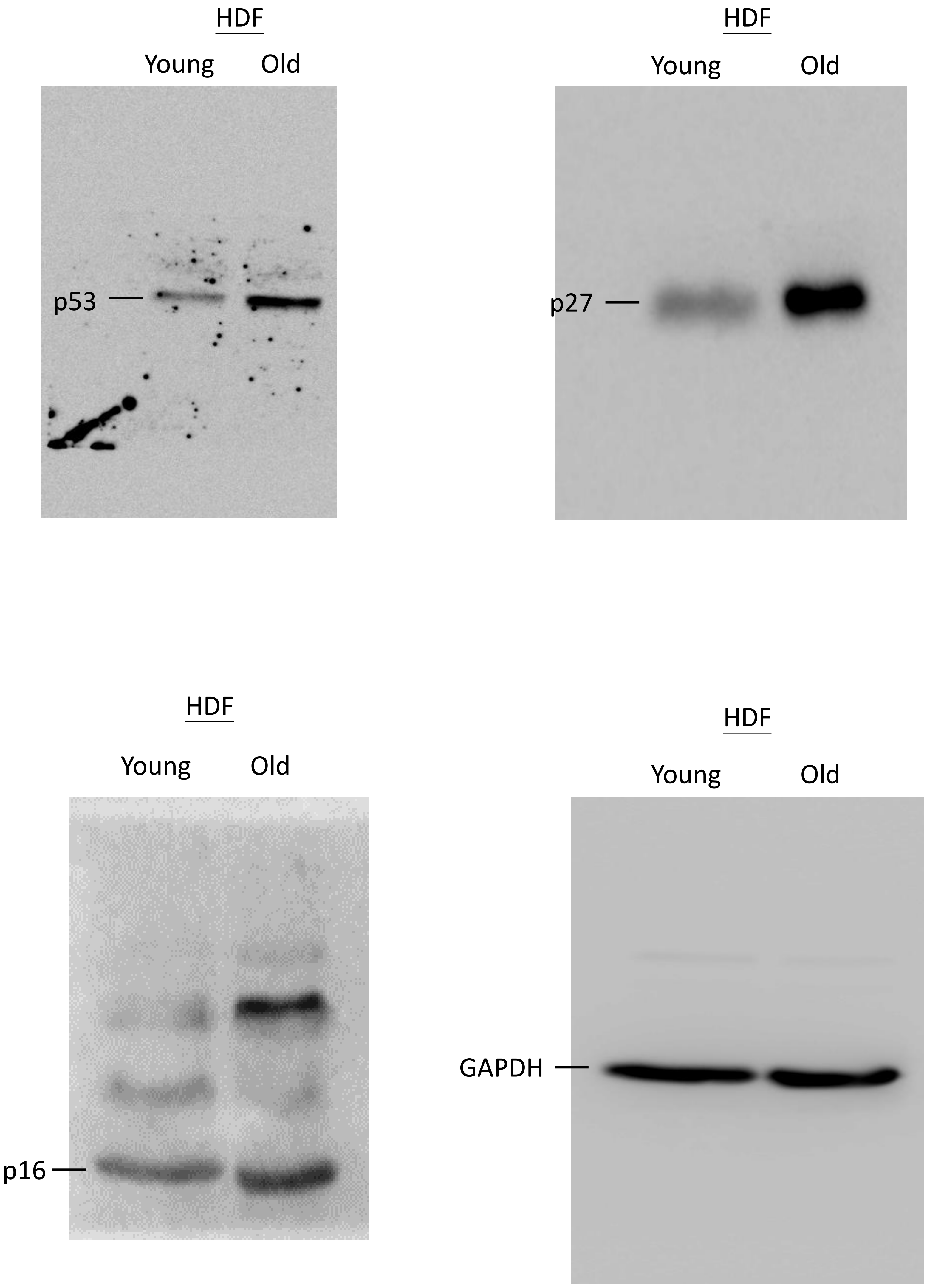

Supplementary Figure 1. Western blot analysis of senescent related proteins in HDFs.
